# Clinical validation of a three-marker methylation panel to detect CIN3+ in vaginal self-samples in the Dutch population-based screening programme

**DOI:** 10.1186/s13148-025-02020-w

**Published:** 2025-11-23

**Authors:** Jolien de Waard, Arkajyoti Bhattacharya, Martine T. de Boer, Bettien M. van Hemel, Martha D. Esajas, Karin M. Vermeulen, Geertruida H. de Bock, Ed Schuuring, G. Bea A. Wisman

**Affiliations:** 1 Department of Gynecologic Oncology, Cancer Research Center Groningen, University of Groningen, University Medical Center Groningen, PO-box 30.001, 9700 RB Groningen, The Netherlands; 2 Department of Medical Oncology, Cancer Research Center Groningen, University of Groningen, University Medical Center Groningen, Groningen, The Netherlands; 3 Department of Pathology, University of Groningen, University Medical Center Groningen, Groningen, The Netherlands; 4 Department of Obstetrics and Gynaecology, University of Groningen, University Medical Center Groningen, Groningen, The Netherlands; 5 Department of Epidemiology, University of Groningen, University Medical Center Groningen, Groningen, The Netherlands

**Keywords:** Cervical cancer screening, DNA methylation, Self-sampling, hrHPV-positive, Clinical validation, Quantitative methylation-specific PCR (QMSP), Cervical intraepithelial neoplasia (CIN)

## Abstract

**Background:**

The use of vaginal self-sampling for cervical cancer screening is promising and increasing. However, triage cytology cannot be performed on vaginal self-sampling material after a high-risk human papilloma virus (hrHPV)-positive result. In our recent discovery study, we identified a three-marker panel with high sensitivity (82%) and specificity (74%) for CIN3 or worse (CIN3+). In the present study, we performed the clinical validation of this three-marker panel using real-world hrHPV-positive vaginal self-samples obtained through the Dutch screening programme.

**Methods:**

The markers *LHX8*,* EPB41L3* and *ANKRD18CP* were analysed using quantitative methylation-specific PCR (QMSP) on a consecutive cohort of hrHPV-positive vaginal self-samples (*n* = 2482: 408 CIN3+ and 2074 <CIN3). Diagnostic performance was assessed using sensitivity and specificity derived from receiver operating characteristics (ROC) analysis.

**Results:**

The three-marker panel showed 73% (298/408) sensitivity and 79% (1640/2071) specificity to detect CIN3+, and identified 96% (21/22) of cervical cancer cases. A scenario analysis was performed on a virtual population of 100 000 hrHPV-positive women using vaginal self-sampling, comparing our methylation triage test with current cytology triage testing. This analysis revealed that more cancers (864 vs. 684 or 770 for 80 or 90% uptake) would be detected with our methylation panel, while referral rates (29% vs. 31% for both 80 and 90% uptake) and detection of CIN3 (72% vs. 68 or 77% for 80 or 90% uptake) would be similar for methylation and cytology.

**Conclusion:**

Compared to cytology triage testing, DNA methylation triage analysis using our three-marker panel offers an appropriate alternative to detect CIN3+ in hrHPV-positive vaginal self-samples. Implementation of the DNA methylation triage test would not only increase cancer detection, but would also eliminate the need for physician visits for cytological triage testing. In addition, it would accelerate referral decisions, ultimately reducing uncertainty and ensuring timely screening completion for all women.

**Supplementary Information:**

The online version contains supplementary material available at 10.1186/s13148-025-02020-w.

## Background

Cervical cancer is the fourth most common cancer type in women worldwide [1]. The implementation of screening programmes has led to a notable decrease in both the occurrence and mortality rates of cervical cancer [2, 3]. Since 2017, the Dutch population-based screening (PBS) programme for cervical cancer is based on primary high-risk human papillomavirus (hrHPV) screening, with cytology triage. This approach was adopted due to limited specificity of hrHPV-testing alone [4, 5]. Women can choose how they would like to participate in the Dutch PBS programme by either going to the general practitioner (GP) for a cervical smear or using a vaginal self-sampling device at home [6].

In the Netherlands, since July 2023, for all women who turn 30 years, their first invitation letter for the PBS programme is accompanied by a home-set package with a vaginal self-sampling device. In addition, women aged 35–60 who do not respond to their invitation letter for a cervical smear by the GP receive a reminder after 12 weeks that includes a self-sampling device [7]. This change has led to a notable increase in self-sampling uptake: ~29 500 women (7% of screening participants) participated by using a self-sampling device in 2017, rising to ~155 800 women by 2023 (44% of screening participants) [8, 9].

Despite the convenience of self-sampling, one of the main disadvantages of using the vaginal self-sampling device is that women with an hrHPV-positive result need to visit the GP for cervical smear collection, because cytology cannot be performed on vaginal self-sampled material. This extra GP visit may be perceived as unwelcome [10], and may contribute to follow-up loss, as 15–20% of the hrHPV-positive women who participated by using the self-sampling device do not visit their GP within 15 months [11, 12]. A molecular triage test, that can be directly applied on the same vaginal self-sampled material, may eliminate the need for this additional GP visit.

Methylation of the promoter region of tumour suppressor genes is associated with cervical carcinogenesis [13–15]. Detection of DNA methylation of host genes is reported to be an objective triage screening method for hrHPV-positive women [16–22]. In addition to being objective, molecular testing for methylation markers offers the advantage of high throughput analysis and can be performed on a cervical smear as well as vaginal self-sampled material [23–25].

In a recently published discovery study, we evaluated 15 previously reported methylation markers in a cohort of hrHPV-positive self-sampled material obtained through the PBS in the North of the Netherlands [22]. The aim was to identify a panel of methylation markers with the highest sensitivity and specificity for the detection of CIN3+ in vaginal self-sampled material. Clustering analysis and model-based recursive partitioning (MOB) revealed a robust three-marker panel *(LHX8*,* EPB41L3* and *ANKRD18CP)* with 82% (79/96) sensitivity and 74% (153/208) specificity for CIN3+ and detection of all cancer cases [22]. Since the discovery study was focused on the identification of the optimal combination of methylation markers to detect CIN3+ rather than on clinical validation, we used a small selected cohort of vaginal self-sampled material, enriched for CIN3+ cases to ensure statistical power (80%). However, the distribution of the various histological subgroups in this selected cohort did not reflect the real-life distribution. Therefore, in the present study, we assessed the clinical validation on a consecutive cohort of hrHPV-positive real-world vaginal self-samples obtained through the PBS in the North of the Netherlands (*n* = 2482). Additionally, using a scenario analysis, we investigated the effect of implementing molecular triage testing compared to the current cytological triage testing in women using vaginal self-sampling.

## Methods

### Study design

To clinically validate the performance of the three marker panel *LHX8*,* EPB41L3* and *ANKRD18CP*, a consecutive cohort of hrHPV-positive real-world vaginal self-samples obtained through the PBS in the North of the Netherlands was used.

The sample size for this study should be at least 2140 hrHPV-positive samples, as the cohort needs to consist of 420 samples of women who have disease (CIN3+) [26]. An a priori power analysis was conducted using G*Power 3.1.9.7 [26] to estimate the required sample size for an exact McNemar test (one-tailed). 90% concordance is assumed, which means a discordance rate of 10% between paired observations. A 5% sensitivity drop was used (sensitivity of the methylation test set at 84% based on test set in discovery study [22] and sensitivity of the cytology test set at 89% [27]), which resulted in an odds ratio of 0.33. With a significance level of α = 0.025 and power of 90%, the analysis indicated that a minimum of 400 samples would be required. With an expected dropout rate of 5% there need to be 420 samples included [22]. In addition, during collection of the 420 CIN3+ samples, simultaneously at least four times more samples of women without disease were collected (*n* = 1680) [28], allowing to determine that specificity of methylation test (set at 71% based on test set in discovery study) [22] is non-inferior to cytology (set at 79%) [27] with >90% power [26]. Sample collection was stopped when there were more than 420 CIN3 + samples in the database, while all <CIN3 samples collected during the same time period were included.

Samples were collected between June 2020 and March 2022 from women (aged 30–63) who participated in the PBS in the North of the Netherlands by using a vaginal self-sampling device (Evalyn Brush, Rovers Medical Devices B.V., Oss, the Netherlands) and had an hrHPV-positive result using the Cobas^®^ 4800 HPV test (Roche Diagnostics, Alameda CA, USA). All consecutive samples were collected during this time period to ensure the distribution of the various histological subgroups reflecting real-world data from women using vaginal self-sampling. Vaginal self-samples of women without complete follow-up in this same period were excluded. Local biobank permission was obtained according to the local and National Institute for Health and the Environment (RIVM) regulations. Histology of the biopsy taken by the gynaecologist was used as the gold standard. Histology and cytology results were retrieved at the nationwide network and registry of histo- and cytopathology in the Netherlands (PALGA Foundation). Histology was categorized as CIN0, CIN1, CIN2, CIN3, and cancer. Women with two consecutive normal cytology results (at primary screening [*t* = 0 months] and ~ 6 months of follow-up screening [control smear], and who were therefore not referred to the gynaecologist for colposcopy) were considered as hrHPV-positive women without disease (i.e. NILM).

### Epitect direct bisulfite treatment

Direct bisulfite conversion was performed using the Epitect Fast LyseAll 200 Bisulphite conversion kit (QIAgen, Hilden, Germany). Cervical cells were collected by taking 500 µl from the PreservCyt collection tube, centrifuged for 10 min at 500 rcf, and the supernatant was removed. In case of visible low cell amounts (no pellet) the volume was increased to 1.5 ml. The direct bisulfite treatment was performed according to manufacturer’s instructions (QIAgen, Hilden, Germany), except that elution was performed in 50 µl Elution Buffer. A pool of leukocyte DNA (1 µg input) from healthy women was used as a quality control to check if the bisulfite conversion was complete (unmethylated control). In addition, in vitro methylated genomic DNA with Sss I (CpG) methyltransferase (New England Biolabs, Beverly, MA, USA) (1 µg input) and HeLa cells (1.25 * 10^5^ cells) were used as a methylated controls. The kit was first validated on a small cohort of cervical samples and vaginal self-samples with different outcomes. This pilot showed agreement in methylation positivity and negativity between our previously used method (salt-chloroform DNA isolation followed by the EZ DNA methylation kit) and the direct bisulfite conversion. In addition, a study showed that direct bisulfite conversion with Epitect and DNA isolation (by a Microlab Star Robotic system) followed by bisulfite treatment (with the EZ DNA Methylation Zymo kit) showed good agreement [29]. Hereafter, the whole cohort of samples was analysed by the direct bisulfite conversion.

### Quantitative methylation specific PCR

QMSP was performed with bisulfite-treated DNA as described previously for the three markers, *ANKRD18CP*,* LHX8* and *EPB41L3* [22]. Each sample was analysed in a 96-well plate using the Abbott m2000rt System (Abbott Molecular Inc., Des Plaines, IL, USA). As a methylation positive control, in vitro methylated genomic DNA with Sss I (CpG) methyltransferase (New England Biolabs, Beverly, MA, USA) was used in each run. H_2_O was used as negative control. All amplification curves were reviewed and scored without knowledge of clinical data. A sample was considered invalid due to too low levels of genomic DNA if the Ct-value for β-actin was ≥ 32. QMSP values (∆Ct values) were adjusted for DNA input by expressing results as ratios between two absolute measurements (Ct value marker – Ct value β-actin). Methylation levels were calculated using the formula 2^−∆Ct * 100. Negative samples (no Ct value before 50 cycles) were assigned a ∆Ct of 30.

### Statistical analysis

Statistical analysis was performed using SPSS software package (SPSS 28, Chicago, IL, USA) and RStudio software (version 1.4.1106). ROC curves were generated based on ∆Ct values to detect CIN2+ and CIN3+ as cut-off, and the AUC was used as a measure of model performance. A Kruskal Wallis test was used to compare several groups. A Mann-Whitney *U* test was performed to identify differences in methylation levels among CIN3+/<CIN3 and CIN2+/<CIN2. Differences in results were considered statistically significant when the *p*-value was < 0.05. Graphical representations were created with GraphPad Prism 9 or RStudio software (version 1.4.1106).

### Model-based recursive partitioning (MOB) to construct a predictive model for the detection of CIN3+

In our previous study, we developed a predictive model for CIN3+ detection using MOB [22]. However, in the current study, the decision tree modelling had to be re-executed due to methodological differences. Specifically, the direct bisulfite treatment used in this study resulted in higher ∆Ct values compared to the previous method that involved DNA isolation followed by bisulfite treatment. This highlights the dependency of the model on the methodology used; models cannot be directly applied across different protocols without recalculating the coefficients. To achieve optimal sensitivity and specificity using the three selected methylation markers, a new predictive model was developed through model-based recursive partitioning. This involved evaluating all seven possible combinations of the three markers. The detailed methodology for model-based recursive partitioning is described in the Supplementary Methods section of de Waard et al. [22].

As described previously [22], the analysis was performed using the mob function from the party package (version 1.3-9) in R. The dataset, consisting of samples and their corresponding ∆Ct values for all tested markers, was divided into a training set (70%, *n* = 1737) and a test set (30%, *n* = 745) using stratified random sampling without replacement based on histological diagnosis. This ensured both sets contained an equal proportion (~ 16%) of CIN3+ samples. All markers were considered as classifiers, and combinations of one to three markers were evaluated as predictors. The cut-off for predicting CIN3+ was determined using the ROC curve on the training set, selecting the threshold closest to the top-left corner. The predicted probability per sample could be calculated by filling in the delta Ct value for the selected methylation marker in the decision tree, and thereafter filling in the delta Ct values for the methylation markers in the logistic regression formula at the associated node.

To assess the robustness of the models, a robustness analysis was conducted by repeating the MOB process 1000 times. In each iteration, 70% of the samples were randomly assigned to the training set, and the remaining 30% to the test set. The criteria for selecting robust models were consistent with those described in our previous work [22].

### Scenario analysis to evaluate the performance of methylation triage compared to cytology

**Fig. 1:**
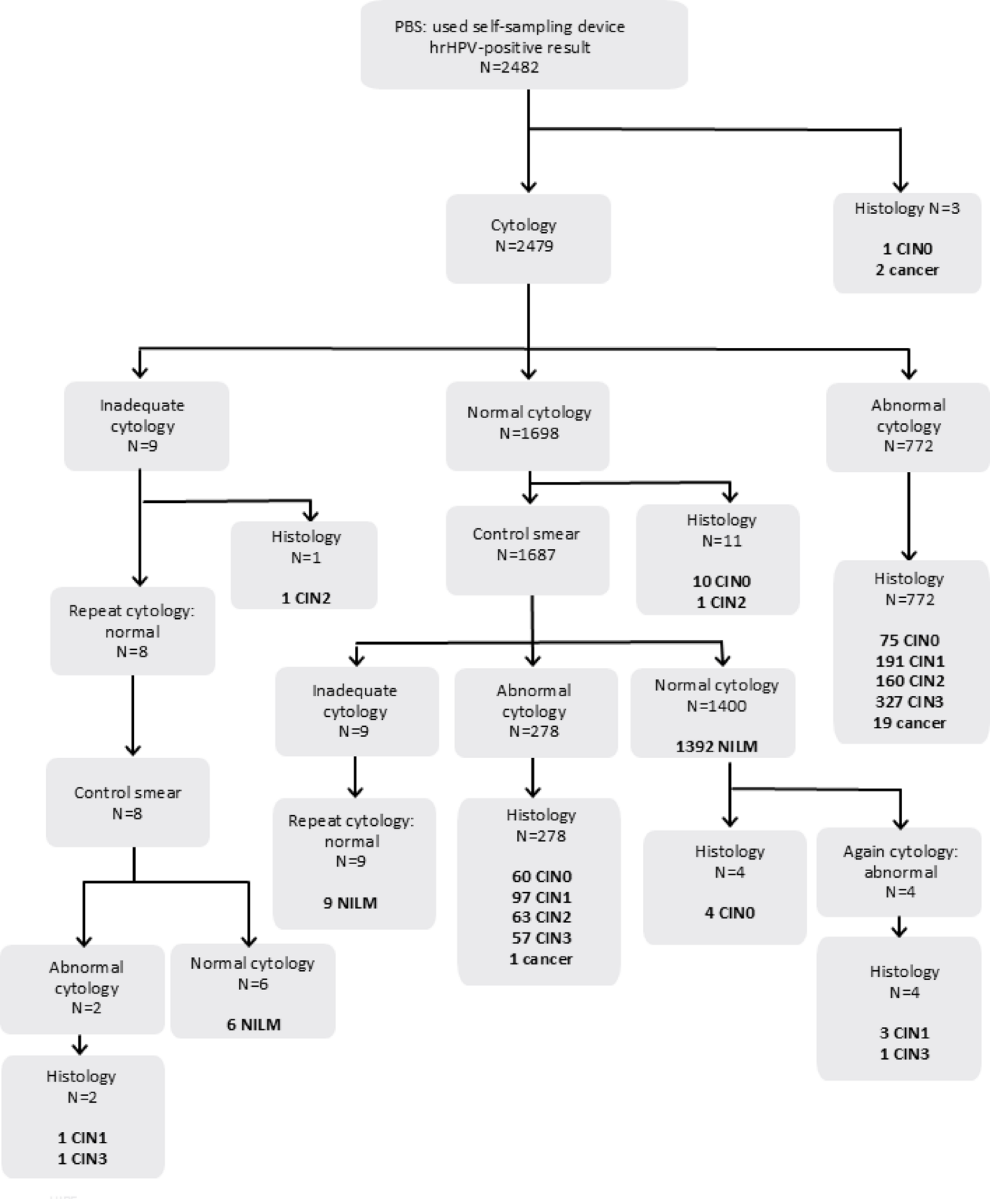
Flowchart of included samples of women with their cytology and histology results. Samples were eligible according to our sample criteria. Samples were collected between June 2020 and March 2022 from women who had participated in the PBS in the North of the Netherlands by using a self-sampling device, had an hrHPV-positive result and a histology result. Sensitivity and specificity of the cytological triage test for the detection of CIN3 + calculated based on this figure were 85% and 79%

To study the effect of the use of the methylation triage test on the number of women screened, referral rate, and the number of missed and detected CIN2+, CIN3+ and cancer cases a scenario analysis was performed in comparison with current cytology triage testing. The scenario analysis was performed on a virtual population of 100 000 hrHPV-positive women who had used a vaginal self-sampling device. Distribution of histology was based on the results of PBS in the North of the Netherlands (Fig. 1). An analysis was performed for three scenarios: 1. Methylation is used as a triage test (meaning that all 100 000 women will be screened). 2a. Cytology is used as a triage test, and 20% of the women will not visit their GP. 2b. Cytology is used as a triage test, and 10% of the women will not visit their GP. In scenario 2, sensitivity and specificity for CIN3+ for cytology were retrieved based on the first round results of cytology on the cohort of vaginal self-samples used in this study, histology results were used as outcome (or only cytology when a women was two times pap1 (Bethesda: NILM)), women that directly went to the gynaecologist without a cytology result were not taken into account (Fig. 1). The percentage of women not going to the GP as used in scenario 2 is based on data of the Dutch PBS [12], where ~20% of the hrHPV-positive women will not visit their GP within the first 15 months. This percentage decreased to 10% after 27 months [12]. The scenario analysis was performed for one screening round, with no follow-up taken into account.

## Results

### Patient samples

In this study, in a total of 3752 hrHPV-positive vaginal self-samples collected between June 2020 and March 2022 were included. Vaginal self-samples of women without complete follow-up in this same period were excluded. Specifically, excluded were cases where only self-sampling was performed (*n* = 182) (so no further follow-up), no control cytology was done (*n* = 486) (so only one cytology result) and of women with pap2+ (Bethesda: ASC-US or higher), but who did not (yet) go to the gynaecologist (*n* = 443). After these exclusions, 2641 women with complete follow-up remained, 16% of whom had CIN3+ (Table S1). The distribution of the diagnosis of the samples was comparable with the Dutch nationwide distribution [27], and mean age of the different histological outcomes was similar (Table S1).

Of the 2641 vaginal self-samples, 2482 samples were valid for methylation analysis based on DNA levels (i.e. < 32 Ct for ACTB). At baseline cytology, 772 women had abnormal cytology, of whom 346 had CIN3+ as outcome (Fig. 1). One thousand six hundred ninety-eight women had a normal cytology result, of whom 1400 had a normal control cytology result. Three women directly underwent histology, two of them had cancer, and one CIN0 (Fig. 1). The cancer cases included in this study consist of one adenocarcinoma, two microinvasive adenocarcinoma, six microinvasive squamous cell carcinoma, and 13 squamous cell carcinoma. The exclusion of the invalid samples (*n* = 159, of whom *n* = 19 were CIN3 and 3 cancer) did not affect the distribution of the histological diagnosis.

### Methylation of *LHX8*,* EPB41L3* and *ANKRD18CP* in hrHPV-positive vaginal self-samples.

To evaluate the diagnostic performance of the markers, methylation levels were first analysed separately. Methylation levels of all three markers increased with the severity of the underlying lesions (*p* < 0.001) (Fig. S1) and discriminated between CIN2+ and <CIN2 (*p* < 0.001), as well as between CIN3+ and <CIN3 (*p* < 0.001). ROC analysis showed an AUC of CIN3+ of 0.81 for *LHX8* (*p* < 0.001, 95% CI 0.79–0.84), 0.76 for *EPB41L3* (*p* < 0.001, 95% CI 0.73–0.79), and 0.69 for *ANKRD18CP* (*p* < 0.001, 95% CI 0.66–0.72 ) (Fig. S2). These results were highly comparable to the analysis of our discovery study (*LHX8*: AUC 0.81, *EPB41L3* of 0.77 and *ANKRD18CP* of 0.65 (all *p* < 0.001)) [22].

### Performance of the three-marker panel

**Fig. 2:**
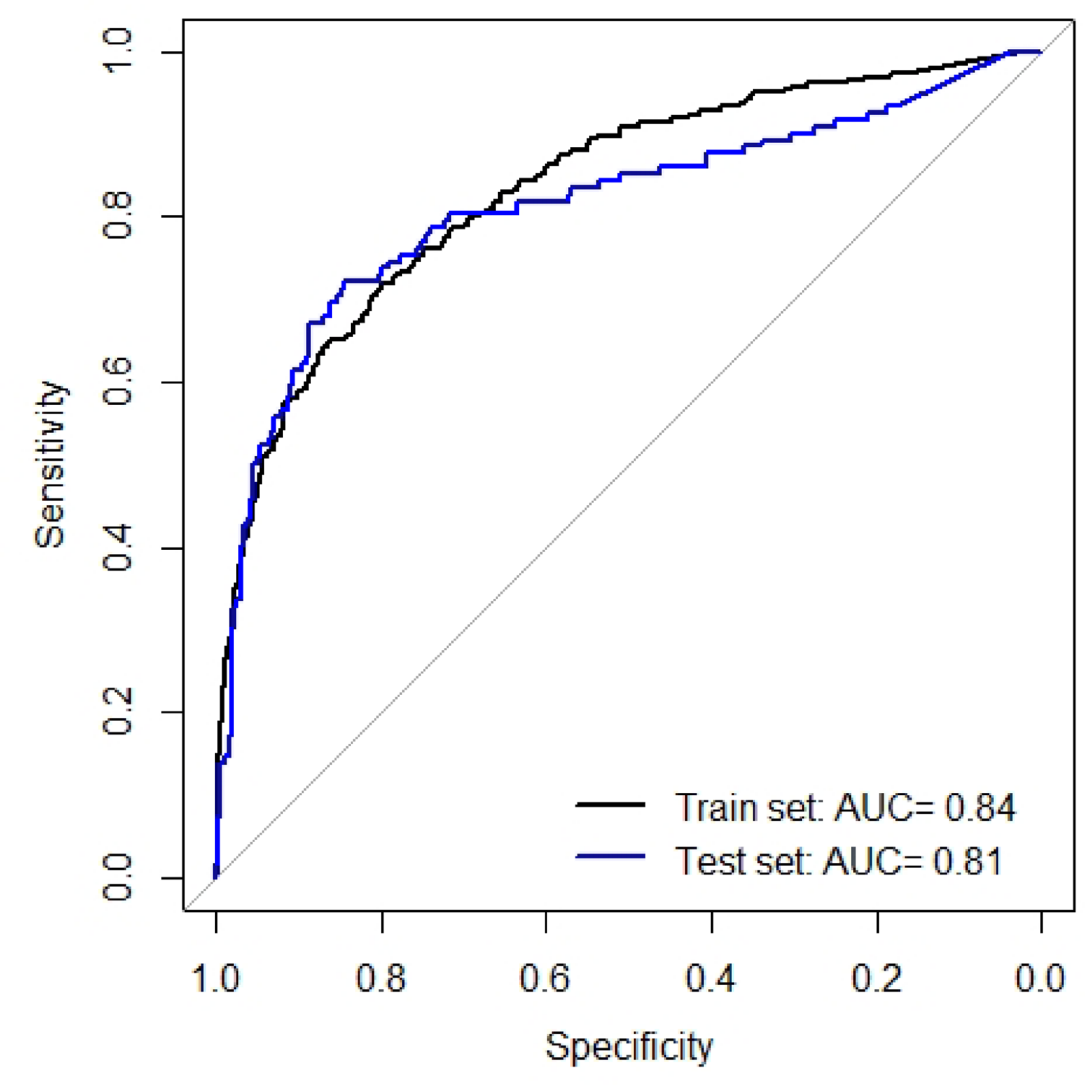
ROC curves of the best performing model to detect CIN3+. The decision tree model consisted of *LHX8* as classifier, and all three markers (*LHX8*,* EPB41L3* and *ANKRD18CP)* as predictors. The ROC curves on the train and the test set with the corresponding AUCs are shown.

**Fig. 3:**
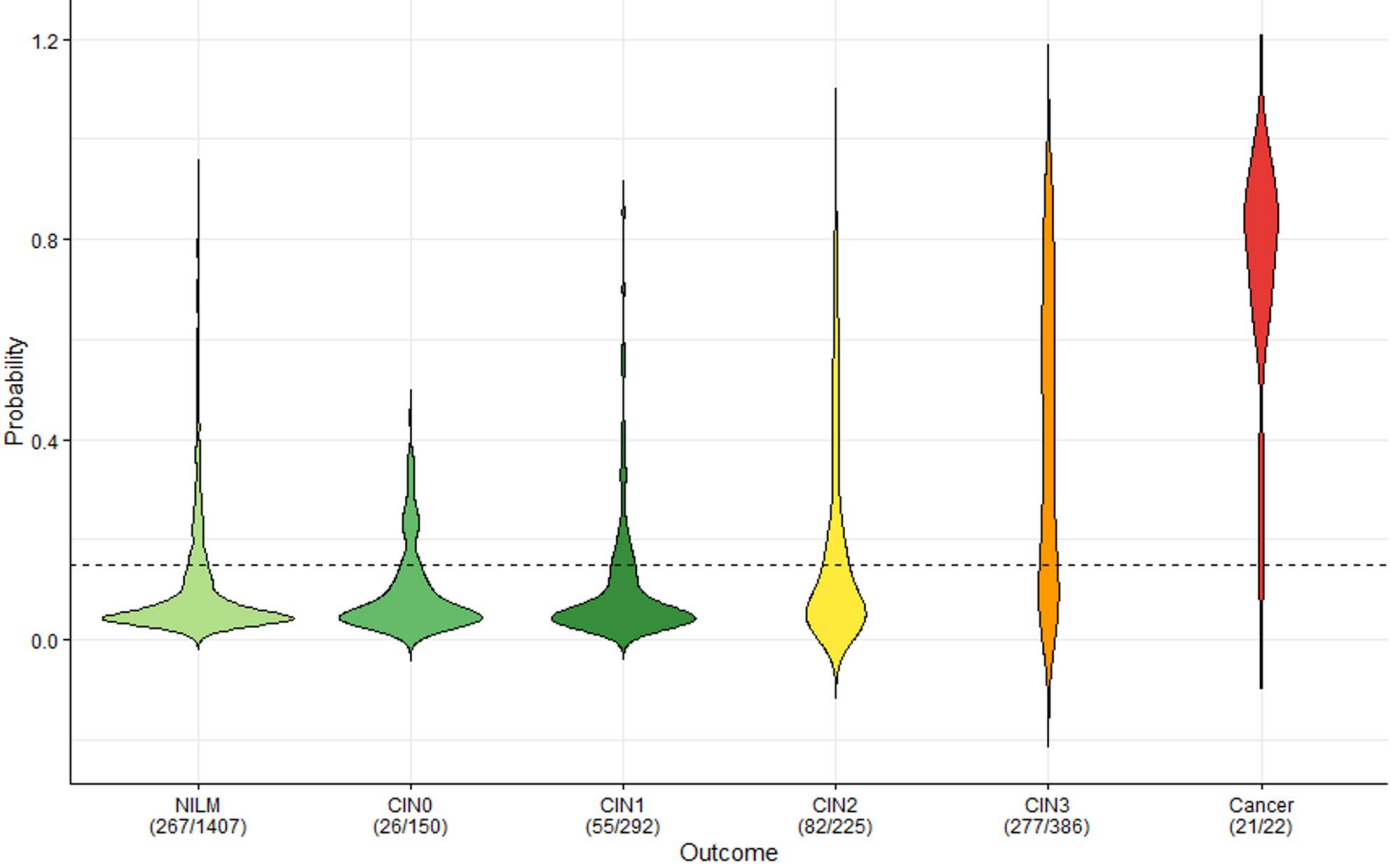
Predicted probability of CIN3+ per histological subgroup. The decision tree model was used to obtain the predicted probability for the different patient samples. The cut-off of the predicted probability to consider a patient CIN3+ based on the ROC curve of the train set and closest top left, is 0.15. The numbers show the amount of cases considered methylation positive relative to the total number of cases in each subgroup.

Due to the use of a different bisulfite treatment method in this study, higher ∆Ct values were observed compared to our previous work. As a result, MOB had to be performed again to develop a predictive model for the optimal detection of CIN3+. Using the methylation markers *LHX8*,* EPB41L3* and *ANKRD18CP* this analysis revealed seven different models, consisting of different combinations of the three markers. All seven models performed well achieving an AUC of > 0.8 in the train set. Additionally, six out of seven models had an AUC of > 0.8 in the test set (Table S2). The model with the best performance based on sensitivity, specificity and the results of the robustness analysis, consisted of *LHX8* as classifier, and all three markers (*LHX8*,* EPB41L3* and *ANKRD18CP)* as predictors (Fig. S3). This model showed an AUC of 0.84 (95% CI 0.81–0.86) in the train set and 0.81 (95% CI 0.76–0.86) in the test set (Fig. 2) and showed high robustness (Table S2). A predicted probability threshold of 0.15 was used to consider a sample as CIN3+. The sensitivity in the train set was 72%, with a specificity of 80%. In the test set, the sensitivity was 75% with 78% specificity. Using this model 96% (21/22) of the cancer cases were predicted as CIN3+, 72% (276/386) of the CIN3 cases were predicted as CIN3 + and 37% (83/225) of the CIN2 cases were predicted as CIN3+ (Fig. 3). If we compare this sensitivity with the sensitivity of cytology triage on the same cohort, non-inferiority for methylation was not obtained. However, non-inferiority was achieved for specificity.

### Scenario analysis

A scenario analysis was performed to provide information on the expected performance of the methylation triage test compared to currently used cytological triage testing in a PBS setting.

For the scenario analysis, we used the sensitivity (73%) and specificity (79%) of the methylation triage test for the detection of CIN3+ (Fig. 3). The sensitivity and specificity of the cytological triage test for the detection of CIN3+ on the same cohort was 85% (342/402) and 79% (1642/2068) (Fig. 1).

Methylation detected 864/900 cancer cases, whereas cytology detected 684 or 770 of the 900 cancer cases for 80 and 90% uptake, respectively (Table 1). However, the number of CIN3 cases detected would have been slightly higher with cytology (when 90% of the women visit their GP), compared to methylation. Moreover, when cytology would be used and 80% of the women visit their GP, there would be fewer CIN3 cases detected than with methylation (Table 1). Although the specificity of both tests was identical, the number of false-positive results would be higher for the methylation analysis. This is because all women (100%) would be screened using methylation, whereas only 80–90% of the women will visit their GP for cytology triage testing. Despite the number of false-positive referrals being slightly higher with methylation analysis than with cytology (21% vs. 16 or 19% for 80 or 90% uptake, respectively), the colposcopy referral rate would be similar for both triage tests (Table 1). Using the methylation triage test, all women will be screened, resulting in the detection of more cancer cases.

**Table 1:** Scenario analysis in a PBS consisting of 100 000 hrHPV-positive vaginal self-samples

	Methylation (scenario 1)	Cytology (uptake of referral: 80%) (scenario 2a)	Cytology (uptake of referral: 90%) (scenario 2b)
Total number of cases screened*	100 000	80 000	90 000
Number of cancer cases detected	864	684	770
% missed cancers**	4	24	14
Number of missed cancers	36	216	130
% CIN3 + detected**	73	68	77
% CIN2 + detected**	60	64	72
% CIN3 detected**	72	68	77
% CIN2 detected**	37	57	64
Number of CIN3 cases detected	11 160	10 540	11 858
Specificity % (CIN3+)	79	79	79
Number of false positive referrals (CIN3+)	17 344	13 751	15 470
Colposcopy referral rate %	29.4	31.2	31.2
PPV %	40.9	44.9	44.9
NPV %	93.8	96.6	96.6

Methylation detected 864/900 cancer cases, whereas cytology detected 770/900 cancer cases. The number of detected CIN3 cases is the highest with cytology, when 90% of the women visit their GP. The complete cohort consisted of: 56 700 NILM cases, 17 800 CIN0/1 cases, 9100 CIN2 cases, 15 500 CIN3 and 900 cancer cases, which is based on the distribution of Fig. 1

*It is assumed that the histology distribution in the lost follow-up women is similar as in the screened population

**Based on whole population (100 000 women)

## Discussion

In this study, we conducted the clinical validation of methylation triage analysis of *LHX8*,* EPB41L3* and *ANKRD18CP* using 2482 consecutive vaginal self-samples that reflect a real-world distribution. Analysis of these 2482 vaginal self-samples revealed sensitivity and specificity for CIN3+ of 73% (298/408) and 79% (1640/2071). Scenario analysis comparing methylation triage testing with currently used cytology triage testing showed very similar performance, despite the lower sensitivity of methylation triage (73%) compared to cytology triage testing (85%). Methylation even detected a substantially high number of cancer cases. Our analysis demonstrated that methylation analysis using these markers is an attractive alternative to replace cytology triage testing, eliminating the need for an extra GP visit for many women worldwide, and shortening the time to referral.

Recently, using 15 previously reported potential methylation markers with high sensitivity and specificity for CIN3+, we reported on the identification of a promising methylation panel (*LHX8*,* EPB41L3* and *ANKRD18CP)* with a sensitivity and specificity of 82% (79/96) and 74% (153/208) for CIN3+ using self-sampling material collected from the PBS [22]. In this discovery study, a selected cohort with 96 CIN3+ cases (91 CIN3 and 5 cancer) and 208 <CIN3 cases (94 NILM, 31 CIN0, 38 CIN1 and 45 CIN2) was used [22]. Since the histology distribution in the discovery study did not represent the real-world distribution in the current Dutch PBS for vaginal self-sampled material, we conducted the clinical validation of methylation analysis in this study using 2482 consecutive vaginal self-samples that reflect a real-world distribution.

In the current study, we used a consecutive series of samples collected over nearly two years, and analysis revealed a notably higher percentage of samples with NILM outcome in particular (57% of the samples versus 30% in our previous study). Despite the difference in composition of the histological groups, methylation analysis demonstrated similar diagnostic performance, with 73% (298/408) sensitivity, and a 79% (1640/2071) specificity vs. 82% (79/96) and 74% (153/208) from the discovery study (both *p* > 0.05).

This is one of the first studies showing the performance of methylation triage markers on a large real-life cohort of vaginal self-samples. The uniqueness of this study lies in using more than 2400 consecutively collected real-world samples of women participating in the PBS in the North of the Netherlands. Using these vaginal self-samples the clinical diagnostic performance of the tested methylation markers is validated without interfering with the regular referral scheme based on cytology triage analysis. No prospective intervention study, with a population-based randomized trial testing the new biomarker compared to the reference standard, was needed. In this way we could determine for the same women the performance of cytology testing versus methylation analysis. Still, such a prospective intervention study with referral based on either cytology (regular screening) or methylation analysis would be interesting to perform.

Currently cytology is used as triage method in the PBS programme in the Netherlands. One of the main disadvantages of cytology is that it cannot be performed on self-sampled material. So, when a women receives an hrHPV-positive result on the self-sampled material, she needs to visit the GP for cytology triage testing, and this extra GP visit may be experienced as unwelcome [10]. The performance of cytology on the cohort of vaginal self-samples used in this study was high with a sensitivity of 85% (342/402) and specificity of 79% (1642/2068) (taking one screening round without follow-up into account). Non-inferiority was not demonstrated for the sensitivity comparing cytology and methylation analysis, while this was obtained for the specificity. However, in the current PBS ~20% of the women will not visit their GP after receiving an hrHPV-positive vaginal self-sampling result within 15 months after the hrHPV result [12], which decreases to ~10% after 27 months [12]. This results in a follow-up loss and a delay in treatment. By using methylation analysis using the same DNA from vaginal self-sampled material as used for primary hrHPV-testing, these additional 10–20% of women will be screened directly. Our scenario analysis showed that methylation analysis detected 72% of the CIN3 cases, while cytology triage testing detected between 68 and 77% CIN3 cases. The main difference found was the detection of cancer cases, as 96% of the cancer cases were detected by using methylation triage testing, whereas cytology triage detected between 76 and 86% of the cancer cases. The detection of more cancer cases by methylation is mainly due to the fact that more women are screened.

This shows that in a group of hrHPV-positive women that do not go to their GP for cytological triage testing (in the current setting), quite a number of cancer cases are missed. These cases would have been identified when applying methylation triage testing on the same vaginal self-sampled DNA used for primary hrHPV testing. On the other hand, despite the finding that the number of false-positive referrals will be slightly higher with methylation triage analysis compared to cytology triage (21% vs. 16–19%), the colposcopy referral rate will be similar for both triage tests.

Yet, a follow-up test for the women with a normal cytology or negative methylation test performed after 6–12 months is not taken into account in the scenario analysis. In the Netherlands, women who receive a normal cytology result after an hrHPV-positive result in the PBS require an extra cervical control smear after 6–12 months. This is because cytology used for one round has been shown to provide insufficient protection [30]. Since methylation has a lower sensitivity for CIN3+ than cytology, an extra control test after 6–12 months may still be necessary. Additional studies are needed to determine the need for retesting hrHPV-positive, methylation-negative cases and the time after which retesting should be performed.

Another aspect to take into account is that the performance of cytology is dependent on the training of the cyto-pathologist, and that cytology in the Netherlands is recognized for its high quality [31]. In countries where the performance of cytology is more challenging, women might benefit from a molecular triage test.

Other studies have also showed the performance of methylation markers on a real-world cohort. Verhoef et al. [32] reported combination of two methylation markers (*LHX8* and *ASCL1)*, on vaginal self-sampling material in a selected real-world cohort (IMPROVE study) and found a 73% sensitivity with a 61% specificity for CIN3+ [32]. The lower specificity compered to our study (79% vs. 61%) will result in more false-positive referrals. Interestingly, one of the methylation markers used in this study [32] was also included in our methylation panel.

Schreiberhuber et al. [33] evaluated three other methylation markers (*DPP6*,* RALYL* and *GSX1)* on a real-world cohort, and showed a 77% sensitivity for CIN2+ with a 77% specificity [33]. However, the exact sensitivity on CIN3+ could not be determined, as high-grade intraepithelial lesion (HSIL) cases were also included. Moreover, this study was performed on GP-collected material and not on vaginal self-sampling material.

An aspect affecting the performance of methylation markers, is that our cohort contained a substantial proportion of samples from women with the outcome NILM (57% of the samples). Histology is not available from these women, as women with two normal cytology results will not go to the gynaecologist for colposcopy/histology, but will instead be reinvited after five years in the screening programme. In our cohort 19% of the NILM cases were methylation positive, which is similar to the methylation positivity observed in the CIN0/1 group, but also to other studies analysing different methylation marker panels [32, 34]. It will be interesting to follow-up these cases in five years to see if these cases were really NILM or that they develop CIN lesions over time and were missed by cytology.

In addition, in our cohort quite some samples (30%) did not have complete follow-up, and were for this reason excluded. This may lead to a bias, although the diagnosis distribution of the samples with complete follow-up was comparable with the nationwide diagnosis distribution.

In our study, 6% of the samples were invalid, which is consistent with our previous and other published reports, regardless of the use of direct or indirect bisulfite treatment [22, 29, 35]. The amount of CIN3+ cases in the invalid samples seems slightly lower than in the complete cohort (13 vs. 16%), which may be caused by the sample collection quality. In this study we did not retest the invalid samples, but in regular screening it would be important to retest the sample or ask the women to provide a new sample, which is general practice in diagnostics.

In this study, where a different bisulfite treatment was used compared to our previous study, comparable AUCs for the three individual markers and performance as a panel were observed. In our previous study we performed DNA isolation followed by bisulfite treatment by the use of the EZ DNA methylation kit (Zymo Research Corp), while in the current study we performed direct bisulfite treatment (Epitect Fast LyseAll Bisulphite conversion kit, Qiagen) on the sample itself. Performing direct bisulfite treatment is advantageous, as this can easily be automated, has high-throughput usage, and is therefore potentially suitable for diagnostic implementation.

## Conclusion

In this study, we validated the sensitivity and specificity of a three-marker methylation panel for the detection of CIN3+ using a cohort of 2482 consecutive vaginal self-samples. This cohort reflected the real-world distribution of various disease groups observed in the Dutch screening population. Our methylation marker panel *LHX8*,* EP41L3* and *ANKRD18CP* showed strong potential as a molecular triage test that can be applied directly to hrHPV-positive vaginal self-samples, offering a viable alternative to cytology. Within the current screening programme, the implementation of the methylation triage test would avoid an extra GP-visit for many women worldwide, resulting in all women being screened, more cancers detected, reduced delay in referral, and more convenience for the participating women.

## Supplementary Information

Below is the link to the electronic supplementary material.

Supplementary Material 1

Supplementary Material 2

## Data Availability

The data generated within this study is shown in Supp. Table 1.
