# Supplementary material for: Clinical validation of a three-marker methylation panel to detect CIN3+ in vaginal self-samples in the Dutch population-based screening programme
Source: Clin Epigenetics. 2025 Nov 23;18:97. doi: 10.1186/s13148-025-02020-w (PMC13202980; doi:10.1186/s13148-025-02020-w)
Supplement: Supplementary file 1 — Supplementary Material 1 [file 13148_2025_2020_MOESM1_ESM.docx]

**Supplementary Material**

**Table S1. Distribution of the different outcomes over the total population.**

| **Outcome** | Number of included women per outcome (with valid result) | Percentage of total included women (percentage of women with valid result) | Mean age of included women (95% CI) | Nationwide distribution [1] |
| --- | --- | --- | --- | --- |
| NILM | 1407 | 57% | 41.2 (40.7-41.7) | 59% |
| CIN0 | 150 | 6% | 41.5 (39.9-43.1) | 22% |
| CIN1 | 292 | 12% | 39.0 (37.9-40.0) |  |
| CIN2 | 225 | 9% | 38.4 (37.2-39.6) | 6% |
| CIN3 | 386 | 15% | 37.6 (36.7-38.4) | 12% |
| Cancer | 22 | 1% | 39.0 (34.5-43.4) | 1% |


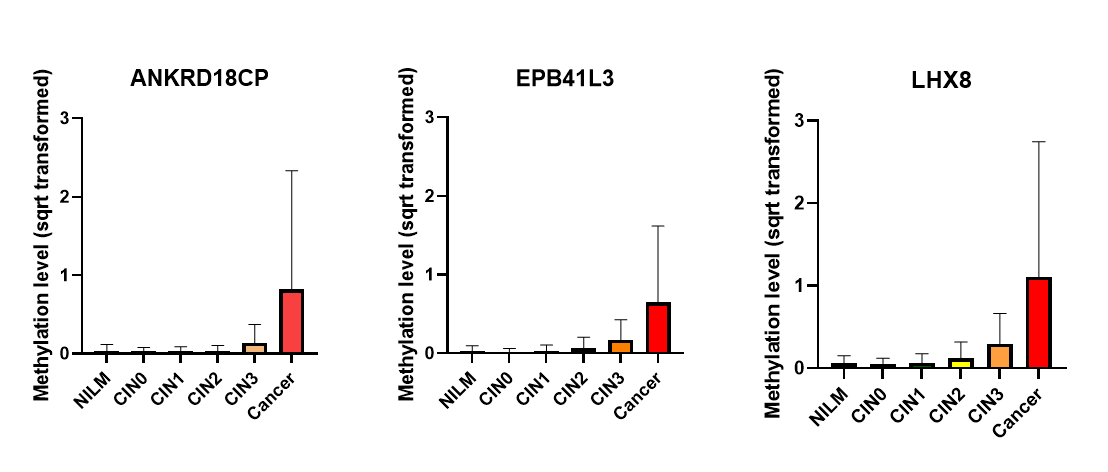


**Figure S1. Methylation levels of the individual methylation markers per outcome.**Methylation levels across histological subgroups (NILM N=1407, CIN0 N=150, CIN1 N=292, CIN2 N=225, CIN3 N=386, cancer N=22) for all the different methylation markers. All markers were discriminative between CIN2+ and <CIN2 (p<0.001) and between CIN3+ and <CIN3 (p<0.001).

**
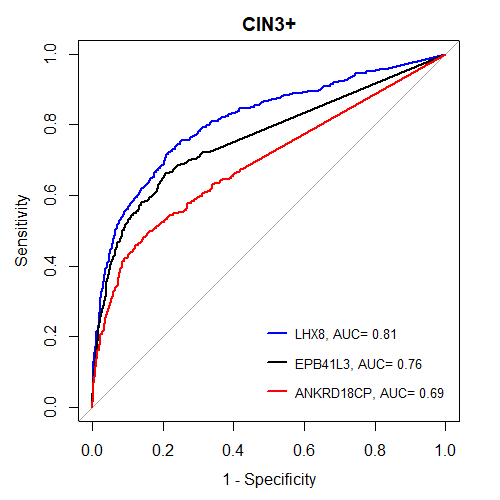
**

**Figure S2.** **ROC curves of the three individual methylation markers for CIN3+.**
ROC curves for ∆Ct values of the three individual methylation markers for the detection of CIN3+ and the corresponding AUCs.

**Table S2. List with possible models with the three selected methylation markers and the robustness score for the classifiers and the predictors.**
The models are ranked based on their robustness scores, for as well the classifiers and the predictors. The model which fulfilled the robustness criteria (score of ≥500) for as well the classifiers and the predictors and mean of standard deviations of the coefficients below 1 is put on top.

| Model | Predictors | Classifiers | AUC train (95% CI) | AUC test (95% CI) | Specificity train | Sensitivity train | Specificity test | Sensitivity test | MCC train | MCC test | Robustness classifier | Mean of sd of coefficients | Robustness predictors |
| --- | --- | --- | --- | --- | --- | --- | --- | --- | --- | --- | --- | --- | --- |
| 1 | *LHX8, EPB41L3, ANKRD18CP* | *LHX8* | 0.84  (0.81-0.86) | 0.81  (0.76-0.86) | 0.80 | 0.72 | 0.78 | 0.75 | 0.43 | 0.43 | 842 | 0.02 | *LHX*8 1000 *EPB41L3* 1000 *ANKRD18CP* 707  Average of predictors: 902 |
| 2 | *LHX8, EPB41L3* | *LHX8* | 0.83  (0.80-0.86) | 0.81 (0.76-0.86) | 0.76 | 0.74 | 0.76 | 0.75 | 0.40 | 0.41 | 791 | 0.03 | *LHX8* 999 *EPB41L3* 999 Average of predictors: 999 |
| 3 | *LHX8* | *EPB41L3, LHX8* | 0.83  (0.80-0.86) | 0.81 (0.76-0.86) | 0.79 | 0.72 | 0.78 | 0.75 | 0.41 | 0.43 | 679 | 0.05 | *LHX8* 1000 |
| 4 | *ANKRD18CP* | *LHX8, EPB41L3* | 0.83  (0.80-0.86) | 0.79 (0.74-0.85) | 0.79 | 0.73 | 0.77 | 0.72 | 0.42 | 0.39 | 282 | 6.32 | *ANKRD18CP* 700 |
| 5 | *EPB41L3, ANKRD18CP* | *LHX8* | 0.83 (0.80-0.86) | 0.80 (0.75-0.85) | 0.77 | 0.74 | 0.75 | 0.72 | 0.41 | 0.37 | 168 | 19.42 | *EPB41L3* 1000  *ANKRD18CP* 263 Average of predictors: 632 |
| 6 | *LHX8, ANKRD18CP* | *EPB41L3, ANKRD18CP, LHX8* | 0.84  (0.81-0.87) | 0.82 (0.78-0.87) | 0.75 | 0.77 | 0.74 | 0.77 | 0.41 | 0.39 | 74 | 0.3 | *LHX8* 972  *ANKRD18CP* 534 Average of predictors: 753 |
| 7 | *EPB41L3* | *LHX8, ANKRD18CP* | 0.83  (0.80-0.86) | 0.80 (0.75-0.85) | 0.78 | 0.73 | 0.77 | 0.75 | 0.41 | 0.41 | 15 | 0.08 | *EPB41L3* 1000 |

**
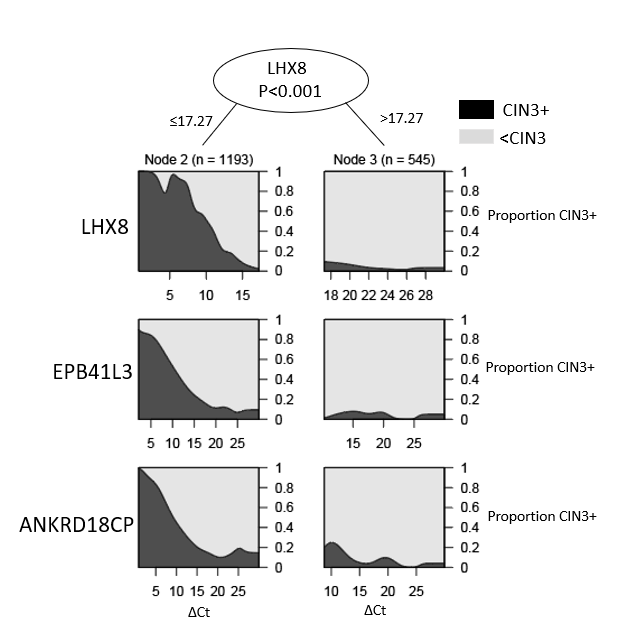
**

**Figure S3.** **The best performing decision tree model to detect CIN3+.**
This model shows whether a sample is regarded as CIN3+/<CIN3+, respectively. The ∆Ct value of the first methylation marker (the classifier, *LHX8*) was used to decide whether the sample had to be placed into the left or the right node. Both nodes consisted of a different linear model. By adding the ∆Ct values of the markers (the predictors, *LHX8, EPB41L3* and *ANKRD18CP*) to this linear model, the odds ratio could be calculated. The graphs show the proportion of CIN3+ lesions at a certain ∆Ct value for each methylation marker.

**References**

1. Inturrisi F, Aitken CA, Melchers WJG, van den Brule AJC, Molijn A, Hinrichs JWJ, et al. Clinical performance of high-risk HPV testing on self-samples versus clinician samples in routine primary HPV screening in the Netherlands: An observational study. The Lancet Regional Health - Europe. 2021;11.
